# Mortality of site-specific cancer in patients with schizophrenia: a systematic review and meta-analysis

**DOI:** 10.1186/s12888-019-2332-z

**Published:** 2019-10-28

**Authors:** Liwei Ni, Jian Wu, Yuming Long, Jialong Tao, Jianhao Xu, Xuya Yuan, Na Yu, Runhong Wu, Yusong Zhang

**Affiliations:** Department of Oncology, The Second Affiliated Hospital of Soochow University, Suzhou, Jiangsu 215004 People’s Republic of China

**Keywords:** Schizophrenia, Cancer, Mortality, Meta-analysis

## Abstract

**Background:**

Numerous studies have reported contradicting results on the relationship between cancer mortality and schizophrenia. Our aim is to quantify the mortality rate of common site-specific cancers among patients with schizophrenia and to synthesize the available research evidence.

**Methods:**

We performed a systemic search of the PubMed, EMBASE and Web of Science databases. Studies reporting the mortality rate of different cancer in patients with schizophrenia were included. A random-effects model was applied to calculate the pooled relative risks (RRs) with 95% confidence intervals (95%CIs).

**Results:**

Seven studies consisting of 1,162,971 participants with schizophrenia were included in this meta-analysis. Data regarding mortality risk of breast, colon, lung and prostate cancer among schizophrenia patients were subjected to quantitative analysis. Pooled results showed significant increases in mortality risk of breast cancer (RR = 1.97, 95%CI 1.38–2.83), lung cancer (RR = 1.93, 95%CI 1.46–2.54) and colon cancer (RR = 1.69, 95%CI 1.60–1.80) in patients with schizophrenia compared with those in the general population or control group. The mortality risk of prostate cancer increased in male patients, although no significant difference was detected (RR = 1.58, 95% CI 0.79–3.15). Increased risks of mortality from lung and colon cancer were observed in female patients (RR = 2.49, 95%CI 2.40–2.59 and RR = 2.42, 95%CI 1.39–4.22, respectively) and elevated risks of mortality from lung and colon cancer in male patients (RR = 2.40, 95%CI 2.30–2.50 and RR = 1.90, 95%CI 1.71–2.11, respectively) were detected.

**Conclusions:**

Individuals with schizophrenia have a significantly high risk of mortality from breast, colon, and lung cancer.

## Background

Schizophrenia is often described as one of the most severe mental disorders. The worldwide prevalence of schizophrenia is approximately 1% [1]. Individuals with schizophrenia have an extraordinarily short life expectancy and die approximately 10–20 years younger than the general population due to the high mortality, mostly resulting from poor medical care, unhealthy lifestyle factors, the side effects of antipsychotic medicine, and suicide [2–4]. The mortality presented by most cancers contributes to the reduced life expectancy of patients with mental illness [5].

As early as the 1990s, individuals with schizophrenia were reported to have a lower or similar risk of cancer mortality [6, 7], a finding that was supported by Giovanni Perini et al. [8]. However, there are a good many studies that demonstrated an increased cancer-related mortality in people with schizophrenia [9–11]. A meta-analysis by Zhou et al. found a high risk of overall cancer mortality after a diagnosis of schizophrenia, but there was highly significant and inexplicable heterogeneity among studies [12].

With respect to specific cancer sites, the results were inconsistent. Tran et al. observed an increased mortality rate from breast and lung cancer in schizophrenia patients [9], whereas Perini et al. described an elevated mortality risk of breast and lung cancer among patients with schizophrenia [8]. These conflicting findings have sparked controversial debates on the association between schizophrenia and the mortality risk of site-specific cancers. Therefore, the present meta-analysis aims to systematically review the available evidence regarding the mortality risk of site-specific cancers in patients with schizophrenia and quantify mortality risk differences by cancer type.

## Methods

### Search strategy

This meta-analysis and systematic review were conducted in accordance with the Meta-analysis of Observational Studies in Epidemiology [13] and the Cochrane Handbook for Systematic Reviews of Interventions guidelines [14]. We systematically searched the PubMed, EMBASE and Web of Science databases for observational studies using the following MeSH terms and textwords: “schizophrenia” [Mesh], “schizophreni*,” “carcinoma” [Mesh], “carcinoma*,” “malignant tumor,” “cancer,” “malignant neoplasm,” “mortality” [Mesh], “mortality*,” “death rate,” and “case fatality rate.” The search was confined to studies on humans published in English up to October 20, 2018. Relevant articles in the reference lists and grey literature were manually screened to update our first literature search.

### Inclusion/exclusion criteria

EndNote X8 software was used to screen relevant articles meeting the criteria for admission and exclude incompatible articles. Studies eligible for inclusion in this meta-analysis must fulfill the following criteria: (1) can be searched for a full text document in English; (2) designed as a retrospective or prospective cohort or follow-up study; (3) the study population already had schizophrenia as a baseline with no history of cancer; (4) the general population or the participants without a diagnosis of schizophrenia formed the control group; and (5) mortality rate [reported as relative risks (RRs) or standard mortality ratios (SMRs) or hazard ratios (HRs) with 95% confidence intervals (95%CIs)] of some site-specific cancers in patients with schizophrenia were documented or could be calculated using data derived from the corresponding article. Studies were excluded if they (1) reported cancer mortality rates without clarification by tumor location and (2) were letters, editorials, abstracts, reviews, case reports, or case-control studies. Two authors (Liwei Ni and Yuming Long) selected the eligible studies independently according to the flow diagram cited from the Preferred Reporting Items for Systematic Reviews and Meta-Analyses (PRISMA) [15] and came to a consensus after cross-checking. Cohen’s kappa statistic was used to measure inter-rater reliability (SPSS version 24. 0, SPSS Inc., Chicago, IL, USA).

### Data extraction

Data were extracted from each study by two authors (Liwei Ni and Yuming Long) independently, and the following variables were obtained: the last name of the first author, publication year, country in which the study was conducted, study design, size of the study population, follow-up period, types of cancer, number of site-specific cancer cases, methods used for confirmation of schizophrenia and cancer death, and mortality risk of site-specific cancers evaluated with 95%CIs. We extracted RRs or SMRs or HRs reflecting the maximum degree of control over potential confounders for preliminary analysis. When necessary, we contacted the primary authors for additional information.

### Quality assessment

The quality of included studies was assessed by the Newcastle–Ottawa Quality Assessment Scale (NOS, scores of 0–9 stars), and studies with NOS ≥ 6 were regarded as high−quality [16]. Two review authors evaluated the risk of bias for each study independently and finally reached a consensus.

### Statistical analysis

All data analyses were performed using the Stata (Version 14.0, StataCorp LP). SMR or HR data were extracted and was transformed into RRs on account of the absolutely low risk of site-specific cancer in patients with schizophrenia, thereby providing a rational basis to ignore differences in measures of mortality rate [17]. RRs with the corresponding 95% CIs for each study were calculated to obtain the pooled estimates. Occasionally, 95%CI data were unavailable and calculated by *P* value and RR [18]. RRs with 95%CIs were calculated in a 2-by-2 contingency table [19] using data derived from the corresponding article.

The chi-squared(χ^2^) tests provided a test of significance for heterogeneity of included studies with *P* < 0.05. The I-squared(I^2^) statistic served as an important means of quantifying the heterogeneity, which could be classified as low, moderate, and high, with upper limits of 25, 50, and 75% for I^2^, respectively [20]. If I^2^ > 50% or P < 0.05, a significant degree of heterogeneity exists among studies. If heterogeneity existed between studies, the random-effects model was applied for meta-analysis of the RR data [20]. Moreover, we adopted a trim-and-fill method for sensitivity analysis to detect the source of heterogeneity. According to the Cochrane handbook, at least 10 studies are required to explore the presence of publication bias by constructing a funnel plot [14]. Thus, publication bias was examined by using Egger’s test [21].

## Results

### Literature search

**Fig. 1:**
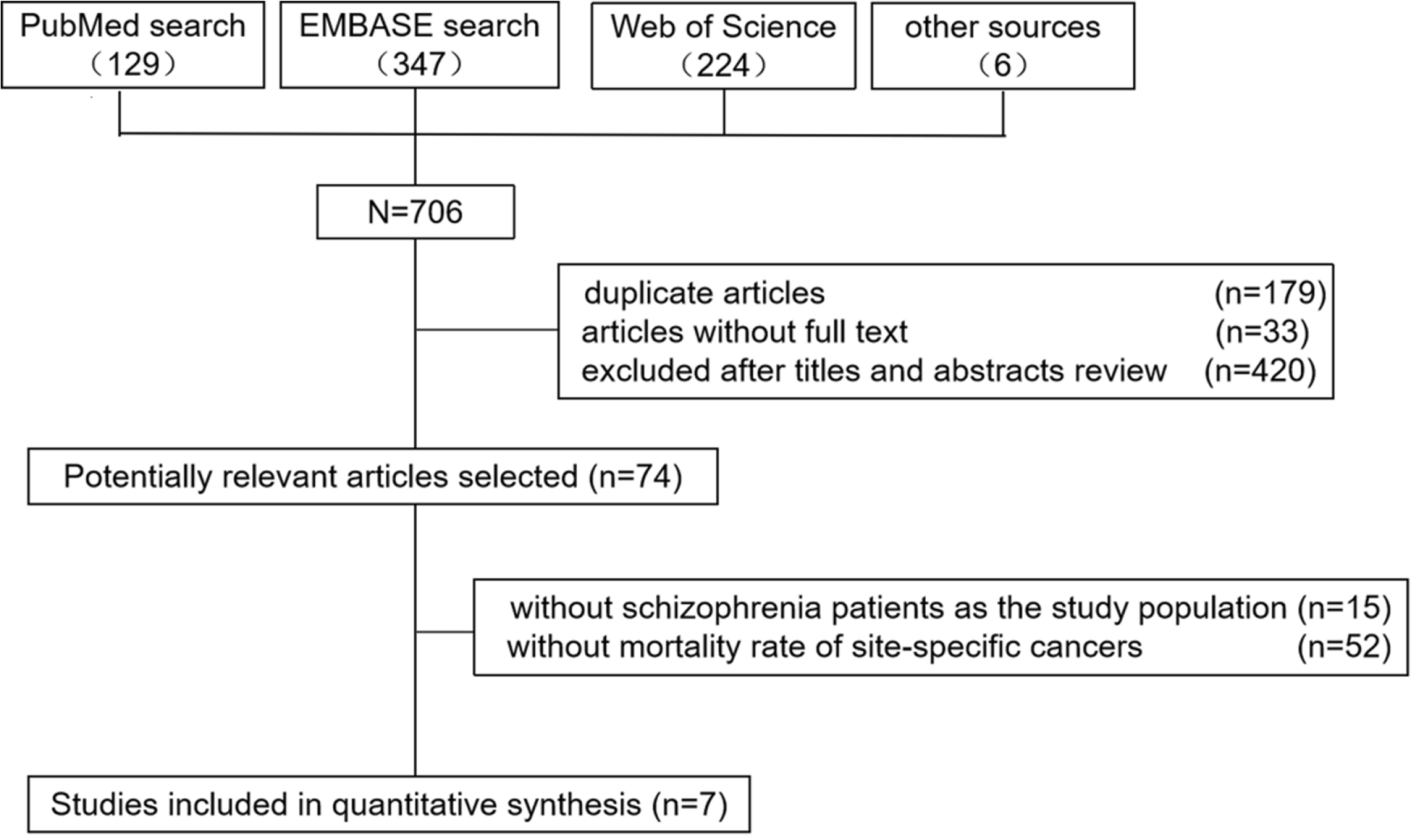
The literature search processes and results

A flow diagram of the literature selection process is shown in Fig. 1. Initially, 706 articles were identified by searching three databases and manually screening the references and grey literature. Then, 179 articles were found to be duplicates and deleted. After reviewing the unique titles and abstracts, 74 records appeared to be potentially relevant for inclusion in the meta-analysis. After further full-text review, 67 studies were excluded because 15 of them did not report schizophrenia patients as the study population, and 52 lacked of data on the mortality rate of any site-specific cancer. Ultimately, seven studies were included in our meta-analysis. The kappa statistic indicated satisfactory agreement between two raters (kappa = 0.82).

### Study characteristics and quality assessment

The characteristics of the included studies are depicted in Table 1. A total of 1,162,971 patients with schizophrenia were enrolled in the seven included studies. Four common cancer types (i.e., breast, colon, lung and prostate cancer) were investigated, considering that a minimum of three data values of mortality rate of a certain cancer were needed for our analysis. Most studies compared cancer mortality in the general population as a control to calculate the RRs [7–10, 22, 23], one study used the cancer mortality rates in a population without schizophrenia to determine RRs [24]. The mortality risk of four selected site-specific cancers was individually reported as SMRs with 95%CIs in major studies and documented as HRs with 95%CIs in only one article [24]. One study did not provide data of 95%CIs corresponding to the SMRs of different cancers [9]. One study reported the data of *P* values and RRs [23]. Thus, we calculated the value of corresponding 95%CIs.

**Table 1:** Characteristics of the included studies for meta-analysis

Author, year(region)	Design	N. of Subjects	Follow-up period	Schizophrenia assessment	Assessent of cancer death	Risk estimate (95%CI)				Adjusted covariates
						Breast cancer	Colon cancer	Lung cancer	Prostate cancer	
Saku et al.,1995, Japan [7]	Prospective cohort study	4980	1948–1985	Hospital records; DSM-IIIR-1987	Japanese family registration system and death certificate	SMR:2.27 (0.27–8.18) in Fp	SMR:3.58 (0.43–12.9) in Fp SMR:0.95 (0.02–5.29) in Mp	SMR:1.68 (0.62–3.66) in Mp	SMR:3.99 (0.1–122.2) in Mp;	Age relative to the general population
Perini et al.,2014, Italy [8]	Retrospective cohort study	695	1982–2006	South Verona Psychiatric Case Register; ICD-10	Mortality Registry of the Local Health District of Verona	SMR: 0.31 (0.03–1.47) in Fp	NR	SMR: 0.24 (0.02–1.14) in Ap	SMR:3.23 (0.64–10.34) in Mp	Age and gender relative to the general population
Tran et al.,2009, France [9]	Prospective cohort study	3470	1993–2003	Questionnaire and hospital records; ICD-10	National death certificate; ICD-9	SMR: 2.8 (1.6–4.9) in Fp	SMR: 5.0 (2.1–12.0) in Fp SMR: 0.7(NS) in Mp SMR: 2.2(NS) in Ap	SMR: 1.5(NS) in Fp SMR: 2.2 (1.6–3.3) in Mp SMR: 2.1 (1.5–3.0) in Ap	SMR: 1.4(NS) in Mp	Age and sex relative to the general population
Olfson et al.,2015,USA [10]	Retrospective cohort study	1,138,853	2001–2007	National Medicaid Analytice Xtract; ICD-10	National Death Index	SMR: 1.60 (1.50–1.70) in Fp	SMR: 1.6 (1.4–1.8) in Fp SMR: 1.9 (1.7–2.1) in Mp SMR: 1.7 (1.6–1.8) in Ap	SMR: 2.5 (2.4–2.6) in Fp SMR: 2.4 (2.3–2.5) in Mp SMR: 2.4 (2.4–2.5) in Ap	NR	Age, race/ethnicity, and geographic region relative to the general population
Brown et al.,2010, UK [22]	Prospective cohort study	370	1981–2006	Hospital records; ICD-9; ICD-10	Death certificate or other official document	SMR: 1.96 (0.54–5.03) in Fp	NR	SMR: 2.65 (1.41–4.53) in Ap	NR	Age relative to the general population
Kredentser et al.,2014,Canada [23]	Retrospective cohort study	9038	1998–2008	Population Health Research Data; ICD-9-CM; ICD-10-CA	ICD-9;ICD-10	NR	NR	RR: 1.45 (1.19–1.76) in A^a^	NR	Age and sex
Crump et al.,2013, Sweden [24]	Retrospective cohort study	8277	2001–2009	Swedish Outpatient Registry and the Swedish Hospital Registry; ICD-10	Swedish Death Registry	HR: 2.58 (1.64–4.05) in Fp	HR: 2.35 (1.22–4.52) in Fp HR: 1.87 (0.89–3.95) in Mp RR: 1.35 (0.83–2.2) in Ap^b^	HR: 2.05 (1.29–3.27) in Fp HR: 1.96 (1.29–2.95) in Mp RR: 1.84 (1.36–2.5) in Ap^b^	HR:1.16 (0.58–2.32) in Mp	Age,marital status, education, employment status, and income,substance use disorder

*Abbreviations*: *N*. of Subjects the number of schizophrenia patients, *DSM* diagnostic and statistical manual of mental disorders, *ICD* International classification of diseases, *SMR* standard mortality ratio, *HR* hazard ratio, *RR* relative risk, *NS* no significance, *CI* confidence interval, *NR* non reported, *Fp* female patients with schizophrenia, *Mp* male patients with schizophrenia, *Ap* all patients with schizophrenia. ^a^95%CI data were calculated by *P* value and RR [18]. ^b^RRs with 95%CIs were calculated in a 2-by-2 contingency table [19] using data derived from an article [24]

Four of the studies provided mortaliry rates of site-specific cancers according to gender [7, 8, 22, 24]. One article reported only on breast cancer mortality in female schizophrenic patients and lung cancer mortality in all participants with schizophrenia [9], one study merely reported mortality data for lung cancer [23], and one study did not provide the mortality data for prostate cancer [10]. The follow-up periods ranged from 7 years to 38 years. Four studies were conducted in European countries [8, 9, 22, 24], two in North America [10, 23], and one in Japan [7]. Most studies used the ICD-9 or ICD-10 and hospital records to assess schizophrenia. Confirmation of cancer death was mainly based on death registry data or death certificate.

All seven cohort studies scored no less than six stars according to the NOS scale, indicating a high level of methodological quality in this meta-analysis (Table 2).

**Table 2:** Quality assessment of eligible studies with Newcastle−Ottawa Scale

First author, year	Representativeness of exposed cohort	Selection of unexposed cohort	Ascertainment of exposure	Outcome of interest not present at start of study	Comparability based on the design or analysis	Ascertainment of outcome	Follow-up long enough for outcomes to occur	Adequacy of followup	Total quality scores
Saku, 1995 [7]	*	–	*	*	*	*	*	–	6
Perini, 2014 [8]	*	–	*	*	*	*	*	–	6
Tran, 2009 [9]	*	–	*	*	*	*	*	–	6
Olfson, 2015 [10]	*	–	*	*	*	*	*	*	7
Brown, 2010 [22]	*	–	*	*	*	*	*	–	6
Kredentser, 2014 [23]	*	–	*	*	*	*	*	–	6
Crump, 2013 [24]	*	*	*	*	*	*	*	–	7

Asterisk represents a point

### Mortality risk of site-specific cancer in schizophrenia

**Fig. 2:**
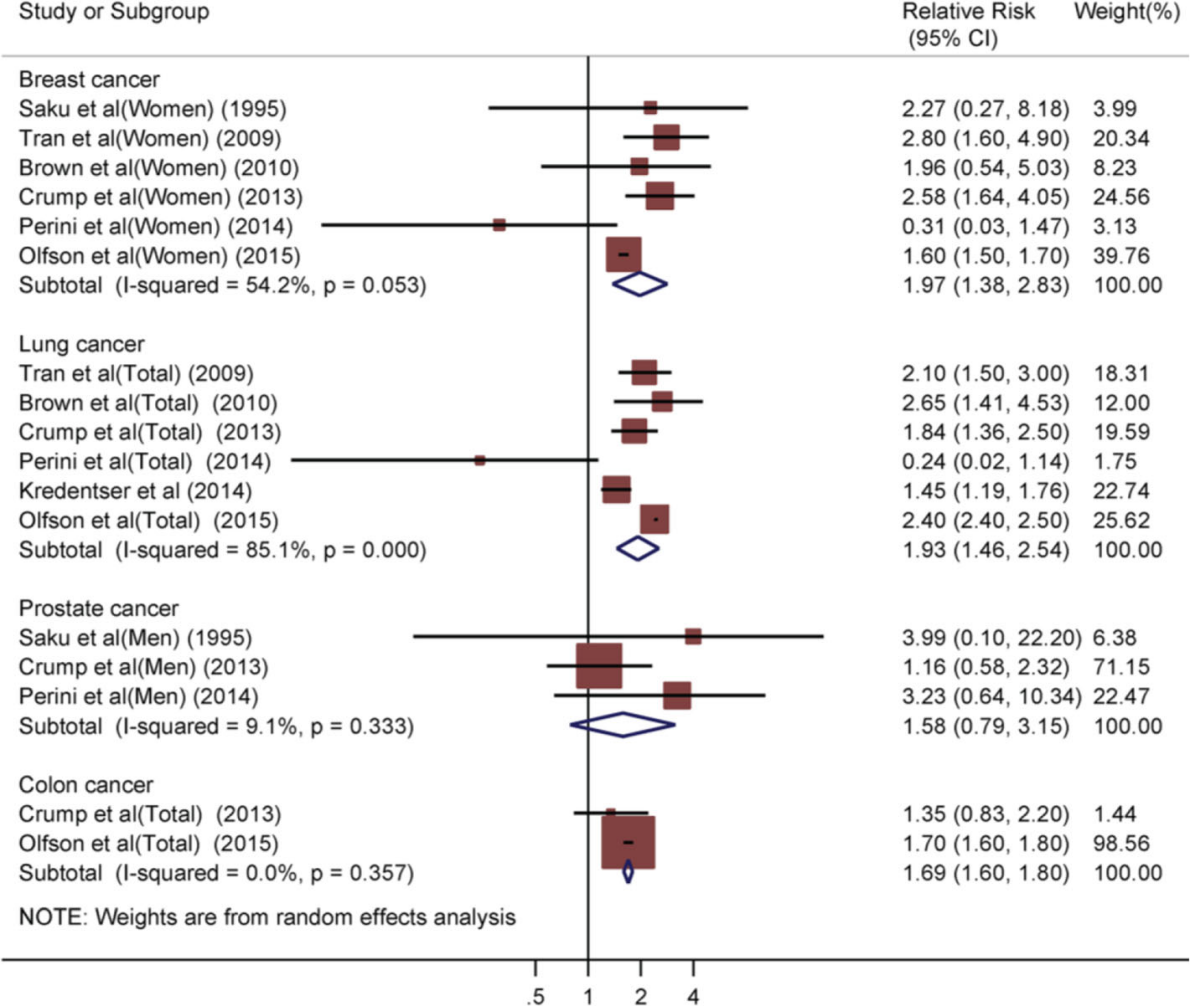
Forest plot of pooled mortality rates of breast cancer, lung cancer, prostate cancer and colon cancer in patients with schizophrenia. Analysis results showed the association between schizophrenia and increased mortality risk of site-specific cancers

Based on each included study’s cancer classifications, we calculated the pooled RRs of four common types of cancer, including prostate (men), breast (women), lung (all patients, men, women), and colon (women, men). The mortality risk of lung and colon cancer was reported in men or women with schizophrenia. We applied the random-effects model to calculate the pooled RRs of site-specific cancers due to the presence of substantial heterogeneity among the included studies (Fig. 2). The pooled RR for breast cancer was 1.97 (95%CI 1.38–2.83, *P* < 0.001), that for lung cancer was 1.93 (95%CI 1.46–2.54, P < 0.001), that for prostate cancer was 1.58 (95%CI 0.79–3.15, *P* = 0.195), and that for colon cancer was 1.69 (95%CI 1.60–1.80, *P* < 0.001). Thus, patients with schizophrenia showed significantly increased mortality risk rates of breast, lung, and colon cancer, but not prostate cancer, compared with the general population or control group.

### Site-specific cancer mortality analyzed according to gender

**Fig. 3:**
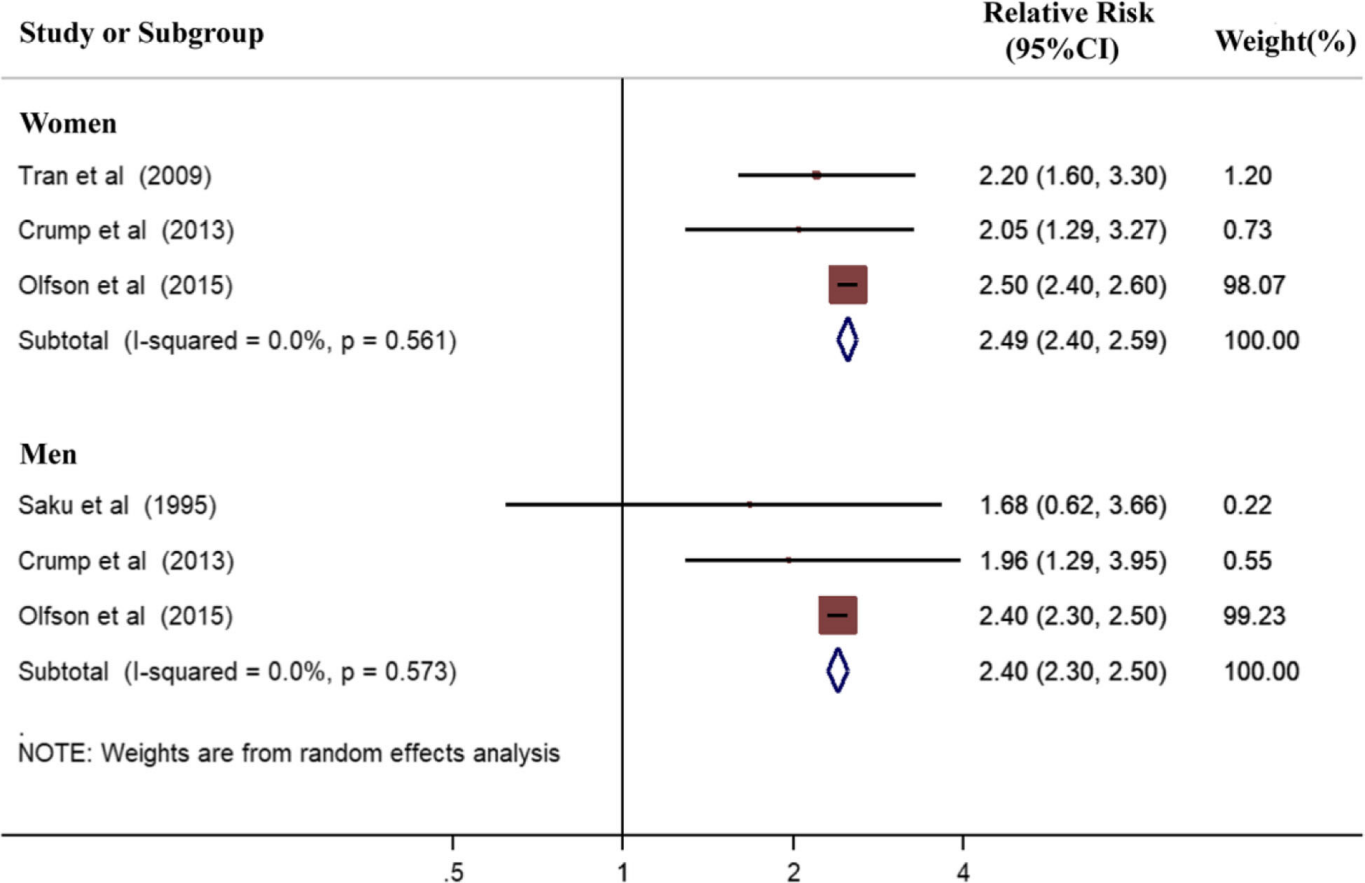
Forest plot of pooled mortality rates of lung cancer stratified by gender in patients with schizophrenia. Analysis results showed the association between schizophrenia and increased mortality risk of lung cancer by gender

**Fig. 4:**
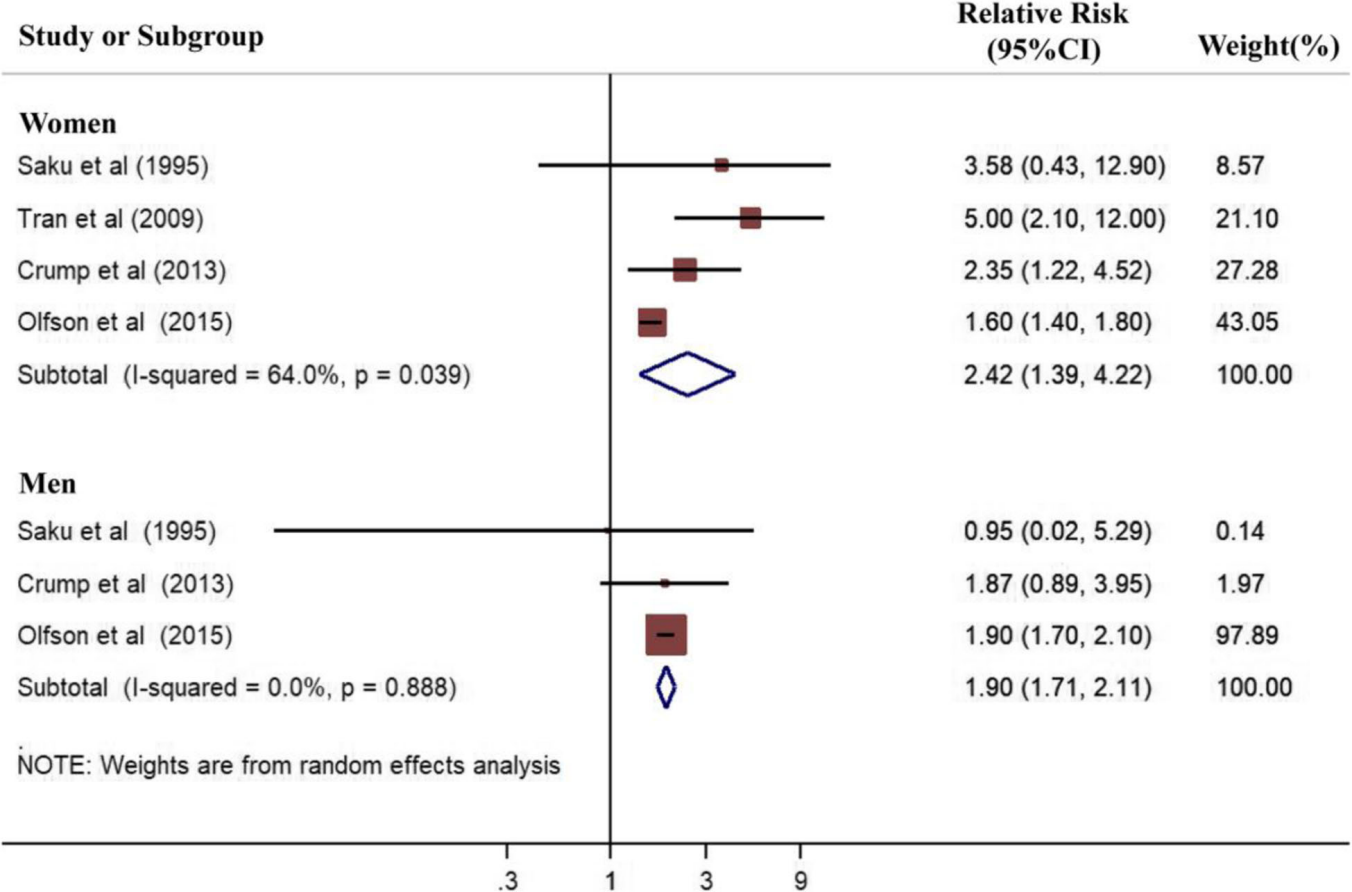
Forest plot of pooled mortality rates of colon cancer stratified by gender in patients with schizophrenia. Analysis results showed the association between schizophrenia and increased mortality risk of colon cancer by gender

Substantial heterogeneity was observed among the mortality rates of lung cancer in all participants with schizophrenia (I^2^ > 50%). We therefore reassessed the results according to gender, which is regarded as a potential confounder (Fig. 3). We also calculated the mortality rate of colon cancer by gender (Fig. 4). For women, the pooled RRs of lung and colon cancer were 2.49 (95%CI 2.40–2.59, *P* < 0.001, Fig. 3) and 2.42 (95%CI 1.39–4.22, *P* = 0.002, Fig. 4), respectively. For men, the pooled RRs of lung and colon cancer were 2.40 (95%CI 2.30–2.50, P < 0.001, Fig. 3) and 1.90 (95%CI 1.71–2.11, P < 0.001, Fig. 4), respectively. Thus, the mortality rate of lung and colon cancer significantly increased in patients with schizophrenia compared with that of the general population or control group regardless of gender.

### Heterogeneity and sensitivity analysis

The χ^2^ test and I-squared statistics were used to evaluate the heterogeneity among studies. The calculated χ^2^ revealed no significant heterogeneity in the mortality rates of site-specific cancer among the included studies (*P* > 0.05, Figs. 2, 3, 4), except for lung cancer in all patients (P < 0.001, Fig 2) and colon cancer in female patients (*P* = 0.039, Fig. 4). The I^2^ statistic indicated high levels of heterogeneity among the cohort studies for breast cancer (I^2^ = 54.2%, Fig. 2) and lung cancer (I^2^ = 85.1%, Fig. 2), as well as low levels of heterogeneity for prostate cancer (I^2^ = 9.1%, Fig. 2). During analysis of cancer mortality rates by gender, significant heterogeneity was observed among the included studies for colon cancer in women (I^2^ = 64.0%, Fig. 4). And statistical heterogeneity did not exist among the studies for colon cancer in men (I^2^ = 0.0%, Fig. 4), and lung cancer in women and men (I^2^ = 0.0%, Fig. 3).

Sensitivity analysis was performed to evaluate the stability of our pooled results in the random-effects model. During multivariate analysis of breast cancer mortality, the results did not significantly flip with a new RR (1.74, 95%CI: 1.24–2.44), which was calculated by the trim-and-fill method (Additional file 1: Figure S1). Each study included in the meta-analysis of breast cancer made no difference to our pooled results in the random-effects model (Additional file 1: Figure S2). During multivariate analysis of lung cancer mortality, the trim-and-fill method was used to obtain a new RR (1.93, 95%CI: 1.46–2.54), which was roughly equivalent to the previous RR (1.97, 95%CI: 1.38–2.83) (Additional file 1: Figure S1). Moreover, the pooled results remained stable regardless of which study in the meta-analysis of lung cancer was omitted (Additional file 1: Figure S2). During multivariate analysis of prostate cancer mortality, although the recalculated RR was similar to the previous value, with no significant difference (Additional file 1: Figure S1), the pooled result became unstable when the study by Crump et al. was excluded (Additional file 1: Figure S2).

### Publication bias

According to the Cochrane handbook, at least 10 studies are required to perform the tests for funnel plot asymmetry efficaciously. Thus, Egger’s test was used to assess the potential publication bias. No evidence of potential publication bias was found among the included studies for breast cancer (*P* = 0.472), lung cancer (*P* = 0.120), and prostate cancer (*P* = 0.299). There were not enough studies for colon cancer to conduct the Egger’s test.

## Discussion

### Main findings and comparison with other studies

The main finding of our study was that patients with schizophrenia have a higher risk of mortality from common site-specific cancers was validated, particularly by a significantly high risk of mortality from breast, lung, and colon cancer. However, the pooled result for prostate cancer was not statistically significant. A previous meta-analysis showed no significant relationships between schizophrenia and breast and lung cancer prevalence; it also revealed that patients with schizophrenia have low incidence risk of colorectal and prostate cancer [25].

These paradoxical findings have directed our attention to the association between schizophrenia and cancer incidence versus mortality risk. A meta-analysis published in 2017 revealed that patients with schizophrenia have a slightly decreased risk of overall cancer incidence compared with the general population [25]. Another meta-analysis based on 16 cohort studies showed a high risk of cancer mortality in individuals with schizophrenia [12]. Zhuo et al. described that female schizophrenia faced a higher incidence of breast cancer than the general population in a meta-analysis [26], whereas Li et al. found a decreased incidence of breast cancer in patients with schizophrenia [25]. Therefore, the association between cancer incidence and mortality risk in patients with schizophrenia remains vague on account of several potential confounding factors, such as gender, ethnicity, genetic background, cancer site, antipsychotic medication use, and cancer care [27, 28].

Evidence supports the possible increased risk of breast cancer in women with schizophrenia due partly to the use of antipsychotic medications [29], some of which may cause hyperprolactinaemia, which, in turn, may contribute to mammary development and breast carcinogenesis in animal and cell experiments [30, 31]. Johnston et al. found that two hyperprolactinemia-inducing antipsychotics, risperidone and pimozide, incite precancerous cells to progress to cancer by activating JAK-STAT5 signaling [31]. Patients with schizophrenia show decreased incidence of prostate cancer [25, 32], which is theoretically related to low testosterone levels suppressed by high prolactin levels on account of antipsychotic drug use [33]. A case–control study revealed that the reduced risk of prostate cancer among patients with schizophrenia is likely associated with long-term treatment of high-dose phenothiazines (primarily chlorpromazine) [34].

Considerable research evidence suggests that genetic factors have been advocated to explain the decreased risk of several types of solid tumors among patients with schizophrenia. P53 and adenomatous polyposis coli polymorphisms may be associated with increased schizophrenia susceptibility and reduced vulnerability to lung and colon cancer in schizophrenia respectively [35–37]. The interaction of tumor suppressor genes TXNIP and AF1q may also contribute to risk for schizophrenia [38]. Wang et al. found that the reduced incidence of prostate cancer in schizophrenia patients may be related to JAZF1 gene mutation [39]. Some of tumor related-genes may be risk factors for the incidence of schizophrenia, and in the meanwhile they may act as protective factors for the development of cancer.

The mortality of different cancers is influenced not only by cancer incidence but also by increased risk of suicide, unhealthy lifestyle, late-stage diagnosis, poor survival after diagnosis, inadequate cancer treatment (e.g., surgery, chemotherapy, radiotherapy, endocrine therapy, and palliative care) [40–42]. Mitchell et al. demonstrated that individuals with schizophrenia are at a high risk of metabolic syndrome (MetS), which is a significant influence upon mortality. Screening for MetS risk factors and taking effective intervention measures, such as exercise, dietary changes and antipsychotic medication management, should be considered [43]. Due to the higher mortality from smoking-related illnesses than the general population, patients with schizophrenia are encouraged to receive pharmacotherapy and behavioral treatments to give up smoking [44]. Moreover, disruptions in the diagnosis and treatment of breast cancer are noticeable for patients with schizophrenia and result in adverse outcomes, including cancer recurrence [45]. Poor diagnostic evaluation and belated stage-appropriate treatment in lung cancer care are common among patients with schizophrenia and lead to poor outcomes [46]. Considering the disparities existent in cancer screening, diagnosis, treatment, and end-of-life care, collaboration between oncologists, psychiatrists, nurses, and other members of the multidisciplinary team is needed to provide high-quality care for patients with schizophrenia.

### Advantages and limitations

Meta-analysis is an important tool to combine the results of included studies that may otherwise be uncertain or imprecise in a single study. The current meta-analysis presents a number of advantages. First, the study population was substantial, which means it has high statistical power. Second, our quantitative assessment was based on cohort studies to better understand the association between schizophrenia and mortality risk of site-specific cancers. Third, no publication biases was detected, which indicates that the pooled results may be stable.

Some limitations must be noted in our meta-analysis. First, on account of the low prevalence of schizophrenia and low cancer mortality in patients with schizophrenia, we ignored imparities in the measures of mortality rate and then calculated pooled RRs, which may produce negligible but inevitable statistical errors [17]. Second, all of the data used for meta-analysis were based on observational studies, and differences in unadjusted covariates are a potential source of bias. Moreover, some of the unrecorded factors may make a difference to the relationship between schizophrenia and mortality risk of site-specific cancers. Third, some data on the mortality risk of the four different cancers surveyed in this meta-analysis were lacking and could not be extracted from the included studies, resulting in outcome selection bias. Finally, although we used a random-effects model to meta-analyze RRs and performed sensitivity analyses to explore uncertainties in the included studies, considerable heterogeneity was still observed in some of our pooled results. Moreover, no adequate baseline information or adjusted confounding factors were available in most of the studies. We therefore cannot carry out meta-regression analyses to further explore the source of heterogeneity in these works.

## Conclusion

Despite some limitations, our meta-analysis demonstrates that patients with schizophrenia have increased risk of mortality from four site-specific cancers, including breast, lung, and colon cancer. Our findings emphasize that clinicians need not only to be aware of the elevated risk of cancer mortality, but also to recognize the differences in site-specific cancer incidence and mortality between schizophrenia patients and the general population. It seems imperative to address the disparities in cancer care and improve survival in people with schizophrenia after a diagnosis of cancer. Multidisciplinary assessment of medical and behavioral conditions is needed. The panel should promote healthy lifestyle intervention and offer individualized psychiatric treatment and anticancer therapy during the whole course of disease.

More research is needed to conduct the site-specific cancer risk assessment in people with schizophrenia. It is preferred to evaluate both incidence and mortality risk in a prospective cohort. To identify a more robust association between site-specific cancer mortality and schizophrenia, further studies need to adequately adjust for confounding factors, such as gender, genetic background, lifestyle, antipsychotic medication use, and cancer care. These cancers could be affected by the hormonally related or metabolic side effects of antipsychotic drugs should be given more attention.

## Supplementary information

**Additional file 1: Figure S1.** Metatrim test of studies included in meta-analysis of breast, lung and prostate cancer. **Figure S2.** Metaninf test of studies included in meta-analysis of breast, lung and prostate cancer.

## Data Availability

All data generated or analysed during this study are included in this published article and its supplementary information files.

## Abbreviations

Ap: All patients with schizophrenia
CIs: Confidence intervals
DSM: Diagnostic and statistical manual of mental disorders
Fp: Female patients with schizophrenia
HRs: Hazard ratios
I^2^: I-squared
ICD: International Classification of Diseases
MetS: Metabolic syndrome
Mp: Male patients with schizophrenia
N. of Subjects: The number of schizophrenia patients
NOS: Newcastle–Ottawa Quality Assessment Scale
NR: Non reported
NS: No significance
PRISMA: Preferred Reporting Items for Systematic Reviews and Meta-Analyses
RRs: Relative risks
SMRs: Standard mortality ratios
χ^2^: chi-squared
