# Supplementary material for: Mortality of site-specific cancer in patients with schizophrenia: a systematic review and meta-analysis
Source: BMC Psychiatry. 2019 Oct 28;19:323. doi: 10.1186/s12888-019-2332-z (PMC6816203; doi:10.1186/s12888-019-2332-z)
Supplement: Supplementary file 1 — Additional file 1: Figure S1. Metatrim test of studies included in meta-analysis of breast, lung and prostate cancer. Figure S2. Metaninf test of studies included in meta-analysis of breast, lung and prostate cancer. [file 12888_2019_2332_MOESM1_ESM.pdf]

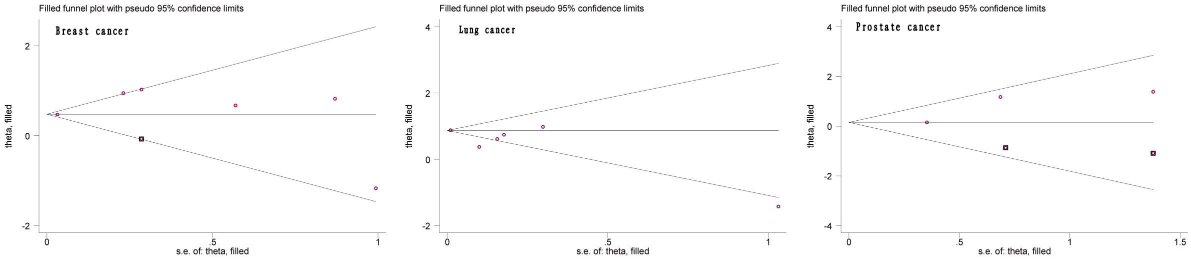


**Fig. S1** Metatrim test of studies included in meta-analysis of breast, lung and prostate cancer

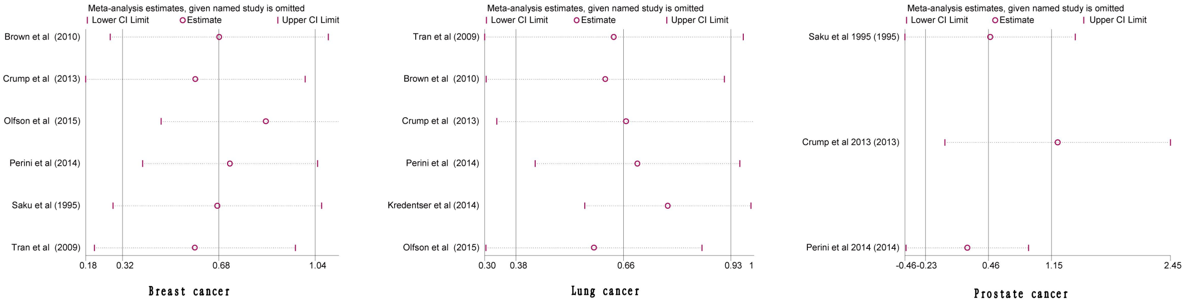


**Fig. S2** Metaninf test of studies included in meta-analysis of breast, lung and prostate cancer
